# Da Vinci robot-assisted thoracoscopic duct ligation for bilateral chylothorax secondary to HIV-related *Penicillium marneffei* infection: A case report

**DOI:** 10.1097/MD.0000000000046317

**Published:** 2026-05-12

**Authors:** Kun Qiao, Shaopeng Li, Pilai Huang, Yanfeng Wang, Zizi Zhou

**Affiliations:** a Department of Thoracic Surgery, National Clinical Research Center for Infectious Disease, Shenzhen Third People’s Hospital, Shenzhen, China; b The Second Affiliated Hospital, School of Medicine, Southern University of Science and Technology, Shenzhen, China.

**Keywords:** bilateral chylothorax, case report, Da Vinci robot-assisted thoracoscopic surgery, HIV, *Penicillium marneffei* infection, thoracic duct ligation

## Abstract

**Rationale:**

Bilateral chylothorax is a rare clinical condition often associated with systemic diseases. This case report presents the successful treatment of bilateral chylothorax caused by human immunodeficiency virus (HIV)-related *Penicillium marneffei* infection using thoracic duct ligation via Da Vinci robot-assisted thoracoscopic surgery.

**Patient concerns:**

A 33-year-old male with HIV and *P marneffei* infection presented with fever, cough, and progressive dyspnea.

**Diagnoses:**

Diagnostic imaging and pleural fluid analysis confirmed bilateral chylothorax.

**Interventions:**

Thoracic duct ligation was performed using the Da Vinci robotic system.

**Outcomes:**

The procedure led to complete resolution of the chylothorax and significant improvement in respiratory symptoms.

**Lessons:**

This case demonstrates the feasibility and safety of robot-assisted thoracoscopic surgery as a minimally invasive and effective approach for managing complex cases of bilateral chylothorax associated with HIV and *P marneffei* infection.

## 1. Introduction

Bilateral chylothorax is a rare clinical condition resulting from injury or obstruction of the thoracic duct, leading to the accumulation of chylous fluid in the pleural cavity. The causes of chylothorax vary and include trauma, tumors, infections, and congenital anomalies.^[1]^ Individuals with human immunodeficiency virus (HIV) are particularly prone to opportunistic infections due to their compromised immune systems, with *Penicillium marneffei* infection being relatively common in Southeast Asia.^[2]^ This fungal infection is characterized by multisystem involvement, including thoracic injury or obstruction, which may result in chylothorax.

Traditional thoracic duct ligation is typically performed using thoracotomy or thoracoscopic surgery. However, these approaches have limitations, including extensive surgical trauma and prolonged recovery times.^[3]^ Recently, robot-assisted thoracoscopic surgery (RATS) has been increasingly utilized in thoracic surgery due to its advantages such as high accuracy, minimal invasiveness, and faster recovery.^[4]^ Despite these benefits, no documented reports exists on its application for bilateral chylothorax caused by HIV-associated *P marneffei* infection. This case report describes the successful treatment of such a case using Da Vinci RATS for thoracic duct ligation.

## 2. Case presentation

**Figure 1.**
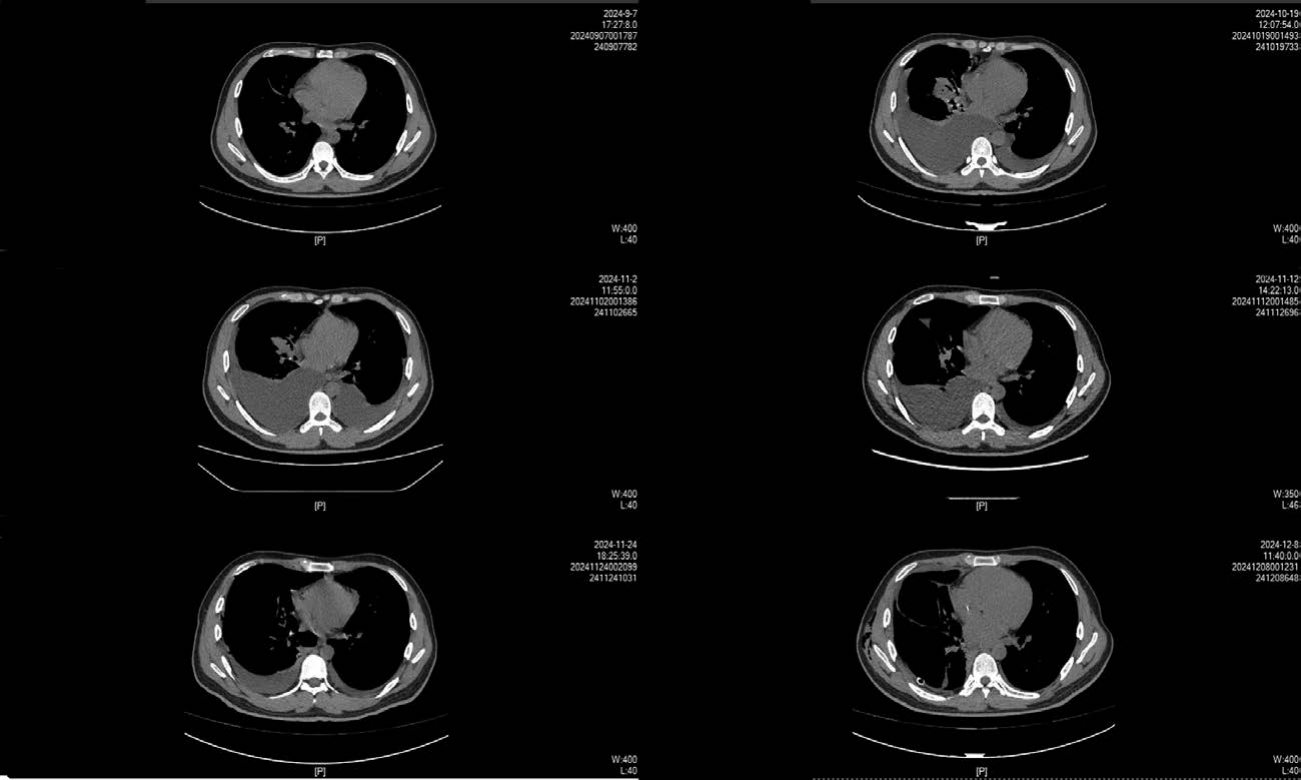
Chest CT scans showing pleural effusion before surgery in different periods and few of them after surgery. CT = computed tomography.

A 33-year-old male with a medical history of HIV and a known *P marneffei* infection was admitted to the hospital in August 2024 with fever, cough, and progressive dyspnea. A chest computed tomography (CT) scan at that time revealed a small right pleural effusion (Fig. 1). The patient was discharged on a regimen of lamivudine–dolutegravir for HIV and voriconazole combined with amphotericin B for antifungal treatment, having declined further diagnostic workup.

On October 19, 2024, a follow-up chest CT showed a significant increase in the pleural effusion (Fig. 1). The patient was readmitted on November 1, 2024, with worsening activity-induced shortness of breath and cough. Chest ultrasound confirmed large bilateral pleural effusions (left: 114 × 97 mm; right: 127 × 112 mm). Percutaneous drainage was performed, and analysis of the pleural fluid confirmed a chylothorax, with persistent daily drainage exceeding 1000 mL.

Despite conservative management (including fasting, parenteral nutrition, somatostatin infusion, and continued anti-infective therapy) the high-volume chyle leak persisted. A flexible thoracoscopic right pleural biopsy performed on November 13, 2024, revealed no pathological abnormalities, and the drainage volume remained unchanged.

**Figure 2.**
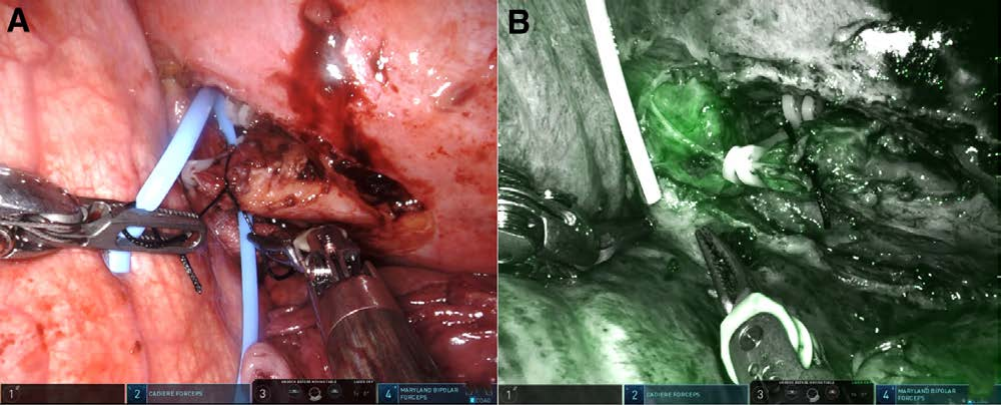
(A, B) Thoracic duct identified through Da Vinci robotic fluorescent mode by right superficial inguinal lymph node injection indocyanine green (ICG) and ligated/sutured by clips and threads through the Da Vinci system.

**Figure 3.**
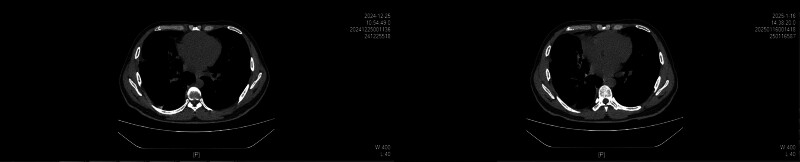
Chest CT scans showing no pleural effusion from 2 follow-up visits after discharge. CT = computed tomography.

Due to the refractory nature of the chylothorax, the patient was transferred to the Thoracic Surgery Department. On December 3, 2024, he underwent a right thoracic duct ligation, which was performed using a Da Vinci RATS (Fig. 2). Postoperatively, bilateral pleural drainage decreased markedly, and the fluid was no longer chylous. Follow-up imaging confirmed the resolution of the effusions. The chest tubes were removed sequentially by December 9, 2024. The patient remained asymptomatic after discharge, with no evidence of effusion recurrence on subsequent chest CT scans (3 weeks and 6 weeks after surgery, Fig. 3).

## 3. Discussion

Individuals with HIV are at high risk of opportunistic infections due to their compromised immune function, with *P marneffei* infection being particularly prevalent in Southeast Asia.^[2]^ This fungal infection can lead to systemic multi-organ involvement, including thoracic duct injury or obstruction, which may result in chylothorax. Additionally, strenuous physical activities such as chest expansion exercises or pull-ups can contribute to lacteal damage, exacerbating the development of chylothorax.^[1]^ In this case, the onset of bilateral chylothorax in the patient may be associated with thoracic duct injury or obstruction caused by *P marneffei* infection.

Thoracic duct ligation via Da Vinci RATS represents a novel and innovative approach for managing bilateral chylothorax in HIV-related *P marneffei* infection. Compared to traditional thoracotomy or thoracoscopic surgery, this minimally invasive technique offers several advantages, including enhanced visualization and precise dissection, which minimize damage to surrounding tissues.^[5]^ It also reduces postoperative pain, shortens hospital stays, and facilitates faster recovery.^[4]^ Additionally, this method leads to shorter operative times, reduced blood loss, and lower complication rates.^[6]^ The Da Vinci robotic system provides superior visualization and dexterity, allowing for precise identification and ligation of the thoracic duct.^[7]^ The successful treatment of this case further validates the feasibility and advantages of RATS in thoracic duct ligation.

Thoracic duct ligation using the Da Vinci system involves several critical steps. The thoracic duct is first identified using robotic fluorescent imaging with indocyanine green injection (dosage: 5 mL, concentration: 2.5 mg/mL) via right superficial inguinal lymph node using B-ultrasound guidance. Comparing to conventional lymphangiography, a radiological examination performed under X-ray imaging after contrast material injection (e.g., iodized oil), it is more simple and time-saving. Once localized, it is meticulously isolated from surrounding tissues. The final step involves applying clips and sutures to ligate the thoracic duct through the Da Vinci system, effectively sealing the duct to prevent further chyle leakage.^[5]^ This precise ligation ensures complete cessation of chyle flow while minimizing damage to adjacent structures.

Despite its benefits, RATS has some limitations. The high cost of the procedure may restrict its widespread adoption, particularly in resource-limited settings.^[8]^ Additionally, the technical demands of this surgery require highly skilled surgeons, which may limit accessibility.^[9,10]^ Further multicenter, large-sample clinical studies are necessary to validate the long-term efficacy and safety of RATS for thoracic duct ligation.

## 4. Conclusion

This case report demonstrates the initial feasibility and successful application of thoracic duct ligation using Da Vinci RATS for a single HIV-positive patient with *P marneffei* infection and bilateral chylothorax. While the outcome is promising and suggests potential benefits over conventional methods in selected cases, broader conclusions regarding superiority are premature. This technique warrants further study to evaluate its generalizability for managing complex thoracic conditions in immunocompromised patients.

## Author contributions

**Conceptualization**: Kun Qiao, Zizi Zhou.

**Data curation**: Kun Qiao, Shaopeng Li, Zizi Zhou.

**Formal analysis**: Shaopeng Li, Pilai Huang, Zizi Zhou.

**Funding acquisition**: Kun Qiao, Zizi Zhou.

**Methodology**: Kun Qiao, Shaopeng Li, Pilai Huang, Yanfeng Wang, Zizi Zhou.

**Resources**: Kun Qiao, Pilai Huang, Yanfeng Wang, Zizi Zhou.

**Visualization**: Yanfeng Wang.

**Writing – original draft**: Zizi Zhou.

**Writing – review & editing**: Zizi Zhou.
